# Effects of implementing free maternity service policy in Kenya: an interrupted time series analysis

**DOI:** 10.1186/s12913-019-4462-x

**Published:** 2019-09-06

**Authors:** Evaline Lang’at, Lillian Mwanri, Marleen Temmerman

**Affiliations:** 1 Department of Health, County Government of Kilifi, P. O Box 9-80108, Kilifi, Kenya; 2 South Australia College of Medicine and Public Health, Flinders University, Flinders University Registry Road, Bedford Park, South Australia 5042 Australia; 3 Director at Centre of Excellence in Women and Child Health, Aga Khan University, Aga Khan University Hospital, P.O. Box 30270-00100, Nairobi, Kenya

**Keywords:** Free maternity service policy, Maternal health service program indicators, Policy implementation, Kenya

## Abstract

**Background:**

Maternal and perinatal mortality is a major public health concern across the globe and more so in low and middle-income countries. In Kenya, more than 6000 maternal deaths, and 35,000 stillbirths occur each year. The Government of Kenya abolished user fee for maternity care under the Free Maternity Service policy, in June of 2013 in all public health facilities, a move to make maternity services accessible and affordable, and to reduce maternal and perinatal mortality.

**Method:**

An observational retrospective study was carried out in 3 counties in Kenya. Six maternal health output indicators were observed monthly, 2 years pre and 2 years post- policy implementation. Data was collected from daily maternity registers in 90 public health facilities across the 3 counties all serving an estimated population of 3 million people. Interrupted Time Series Analysis (ITSA) with a single group was used to assess the effects of the policy. Standard linear regression using generalized least squares (gls) model, was used to run the results for each of the six variables of interest. Absolute and relative changes were calculated using the gls model coefficients.

**Results:**

Significant sustained increase of 89, 97, and 98% was observed in the antenatal care visits, health facility deliveries, and live births respectively, after the policy implementation. An immediate and significant increase of 27% was also noted for those women who received Emergency Obstetric Care (EmONC) services in either the level 5, 4 and 3 health facilities. No significant changes were observed in the stillbirth rate and caesarean section rate following policy implementation.

**Conclusion:**

After 2 years of implementing the Free Maternity Service policy in Kenya, immediate and sustained increase in the use of skilled care during pregnancy and childbirth was observed. The study suggest that hospital cost is a major expense incurred by most women and their families whilst seeking maternity care services and a barrier to maternity care utilization. Overall, Free Maternity Service policy, as a health financing strategy, has exhibited the potential of realizing the full beneficial effects of maternal morbidity and mortality reduction by increasing access to skilled care.

## Background

Maternal and perinatal mortality remains a major public health concern globally with more than 289,000 maternal deaths, 2.6 million stillbirths and 2.7 million neonatal deaths occurring each year [1]. In Kenya the current Maternal Mortality Ratio (MMR) of 362 maternal deaths per 100,000 live births, and the still birth rate of 23 deaths per 1000 live births is far below the target of 147 maternal mortality per 100,000 live births and 12 stillbirths per 1000 live births respectively [2]. The Kenyan government has made significant and purposeful efforts geared towards improving the lives of women over the years and more recently, in June of 2013, Kenya declared maternity services free of charge, in all public health institutions across the country, a move that makes access to quality maternal health care possible for all women in the country [3].

Delivery by skilled health personnel has been established as an effective approach in reducing the risk of maternal and perinatal morbidity and mortality [4] however its utilisation is much less common among the women in Kenya with only 61.8% of deliveries being attended by a skilled provider [5]. High cost of seeking maternity care has been associated with the low utilisation of skilled care during pregnancy and child birth [6].

Fee exemption on maternity care has been implemented in various parts of Africa and Asia where increase in access to maternity care by women of different socioeconomic status, [7] as well as increase in the number women seeking antenatal care services, [8] increase in the number of institutional deliveries, [9, 10] and increase in the facility based caesarean birth rates were observed [11, 12]. In Kenya, studies exploring the implementation process of the Free Maternity Service policy [13–15] highlighted several challenges such as, delayed reimbursements by the government for services provided, recurrent stock-outs of drugs and supplies, reduced staff motivation due to increased workload not matched with increase in health workforce and disruption of the referral system.

### Implementation of the free maternity service policy in Kenya

**Fig. 1:**
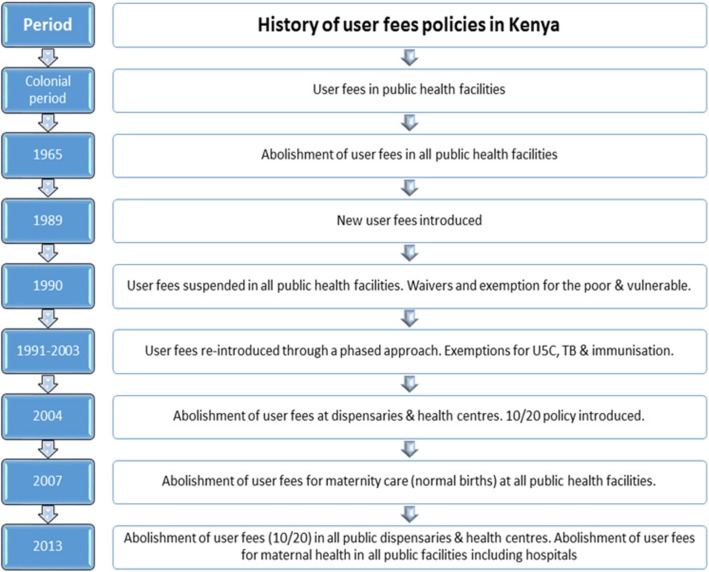
History of user fees policies in Kenya. The figure provides a summarized snapshot of the user fee implementation in Kenya from the colonial period to the year 2013. Sourced from Pyone, T., H. Smith, and N.v.d. Broek, Implementation of the free maternity services policy and its implications for health system governance in Kenya. BMJ Global Health, 2017. 2.

User fee on health care services was introduced in Kenya in 1989, where the fee was to support general operations and management of public health facilities. The introduction of these fees was criticised as a move that would advance social exclusion and inequity in accessing health care services [16] leading to its suspension in the year 1990. Owing to the economic limits within the country, user fees on health care services was later re-introduced in 1991 with exemptions applicable to all children under 5 including immunization charges, treatment for malaria, tuberculosis and sexually transmitted diseases [14, 15, 17]. (Fig. 1).

Later in 2004, the government adopted the 10/20 policy where only a minimal fee of KES 10 and 20 for registration was charged at the public dispensaries and health centers respectively. In a move to promote social protection, maternity services were declared free of charge, in all public health institutions, by the newly elected government on the 1st of June 2013. The Free Maternity Service Policy implementation was immediate after its pronouncement and a follow through on actual implementation process was made by the Ministry of Health (MoH) by way of memos and circulars [14, 15]. The mechanism of its implementation was such that all cost (registration, drugs, services and food) attached to services provided during pregnancy, delivery (whether normal delivery or caesarean births) and postnatally in any of the public health facilities, including hospitals, were transferred from the patient to the government where the government would then compensate these health institutions based on a fixed rate set by the national government [3].

This study thus sought to measure the effects of the Free Maternity Service Policy on utilisation, access and quality of care including the assessment of any adverse effects, such as overmedicalization with cesarean section, that may have resulted from the implementation of the policy.

## Methods

### Study setting

Kenya is governed by 47 semi-autonomous government (counties) and one national government [18]. In Kenya, the health sector comprises the public health system, accounting for 49% of all the health institutions with the major players being the Ministry of Health and parastatal organization [19].

This study took place in three counties, Turkana, Wajir, and Kilifi, all of which serve an estimated population of 3million [19] of which 23% comprise of women of reproductive ages [2]. These counties also rank among the top 15 counties that contribute to the country’s maternal and perinatal death burden [20] and represent 3 of the larger 8 regions in Kenya, namely coast, the rift valley, and north eastern regions [2].

### Study design and sample

An observational retrospective study was conducted in the 3 counties. Owing to logistical and feasibility reasons, 127 public health institutions out of the total 267 in the 3 counties [21] were purposively sampled. However, owing to data quality issues only 90 of these facilities with no missing data were included in the study. The other 37 health facilities were completely deleted from the analysis (Table 1).

**Table 1:** Sampled public health facilities by level of care as per the Kenya Health Master Facility Lists (KHMFL) 2017)

County	County Referral Hospital	Level 4-Sub County Hospital	Level 3-Health Centre	Level 2-Dispensary	Total
Kilifi	1	4	12	11	28
Turkana	1	6	7	20	34
Wajir	1	4	10	13	28
Total	3	14	29	44	90

Variables and information source

Six variables were used to measure the effects of Free Maternity Service Policy as indicated in Table 2 below. These variables were selected as they describe the intermediate output of any maternal health program and service and offers a guide as to whether these programs and services can achieve the overall goal of Maternal Mortality Ratio (MMR) reduction [22, 23].

**Table 2:** Description of the variables used to measure effects of the Free Maternity Service Policy

Indicator	Use of the indicator in the evaluation	Data requirements	Data source
Number of women with at least one antenatal care visit	This indicator will give information about the level of utilisation of antenatal care by pregnant women	The number of women presenting in maternity with at least one or more antenatal care visits during the evaluation period	MOH 333-maternity register
Number of births attended by skilled birth attendants,	The indicator will give information about the utilisation of health facilities by women during birth.	The number of all deliveries attended by skilled birth attendant (doctors, nurses or midwives) during the evaluation period.	MOH 333-maternity register
Number of pregnant women identified with obstetric complication (antepartum and postpartum hemorrhage, preeclampsia, eclampsia, prolonged labour and rupture uterus) and attended	The indicator will give information about the utilisation and accessibility of health facilities.	The number of all pregnant women identified with obstetric complication and attended in level 3, 4, and 5 health facilities during the evaluation period.	MOH 333-maternity register
Caesarean-sections as a proportion of all births.	The indicator will give information about the utilisation of services. (minimum 5%, maximum15%)	The numerator is the number of caesarean sections performed during the evaluation period. The denominator is the total number of births recorded during the evaluation period.	MOH 333-maternity register
Number of livebirths	The indicator will give information about the utilisation of health facilities for childbirth.	The number of live births recorded in the sampled facilities during the evaluation period.	MOH 333-maternity register
Still birth rate	The indicator will be used as a proxy measure of quality of maternal health care.	The nominator is the number of stillbirths (fetal deaths of 28 or more weeks) that occurred during the during the evaluation period. The denominator is number of births (live births + stillbirths) recorded during the evaluation period.	MOH 333-maternity register.

All six variables used in the study included those required for reporting by all the health care facilities in Kenya. For purposes of assuring data quality the data was extracted from the facility’s daily maternity register using a standard template designed and piloted in one of the health facilities by EL as opposed to using the District Health Information Systems (DHIS2) system, a database for reporting and storing health information [24]. The data collectors were trained on the tool and the data variables from the patient records were compared across the 3 standard MoH registers, that is the MoH 333 maternity register, the MoH 711 summary tools and the DHIS 2 system. In the data we checked for inconsistency and missing data across all the registers.

### Statistical analysis

**Fig. 2:**
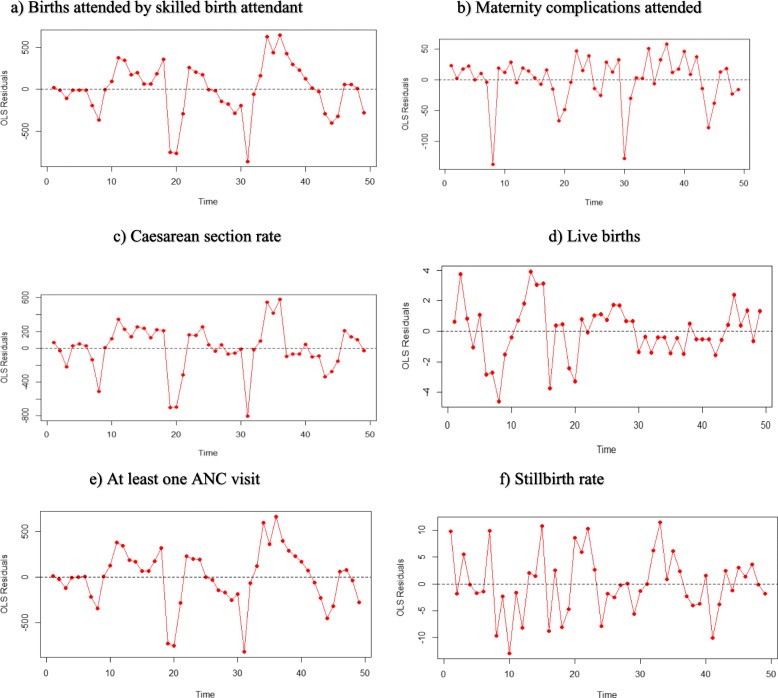
Residuals from the OLS regression in the variables of interest. **a** Births attended by skilled birth attendant. **b** Maternity complications attended. **c** Caesarean section rate. **d** Live births. **e** At least one ANC visit. **f** Stillbirth rate

**Fig. 3:**
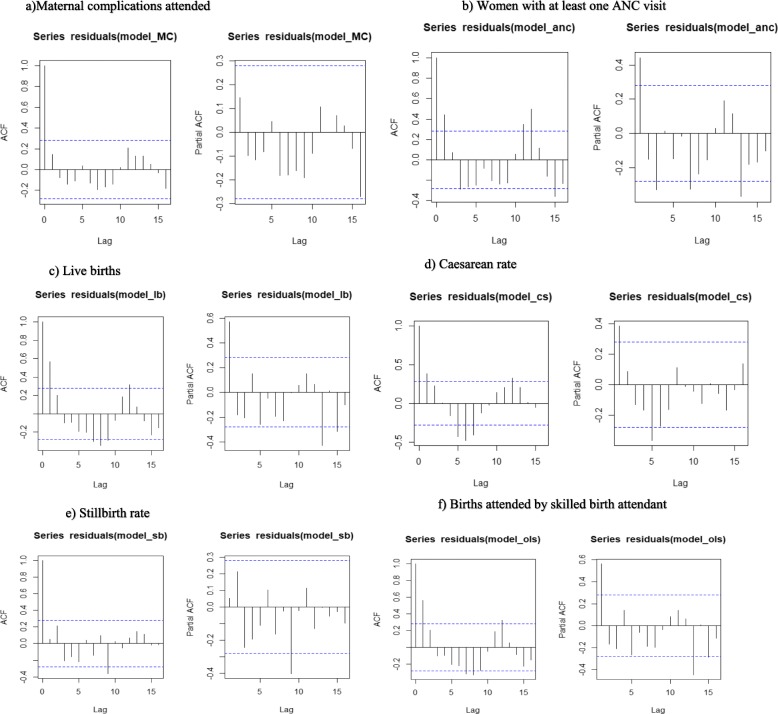
Autocorrelation and partial autocorrelation function of the residuals in the variables of interest. **a** Maternal complications attended. **b** Women with at least one ANC visit. **c** Live births. **d** Caesarean rate. **e** Stillbirth rate. **f** Births attended by skilled birth attendant

The Free Maternity Service Policy having been introduced to the entire national population, use of experimental design to determine effect is not fitting. Interrupted Time Series Analysis (ITSA), a strong longitudinal quasi-experimental design [25–29] with a single group was used to assess the effects of the Free Maternity Service Policy to maternal health [25–28, 30]. The six maternal health output indicators were observed 24 months pre (June 2011–May 2013) and 25 months post (June 2013–June 2015) free maternity service policy implementation, giving a total of 49 observations. Visual inspection of the data was done to check for any wild points, linear trends, and data quality issues. Ordinary least squares (OLS) regression model with a time series specification (an intercept term, a trend term, a level change, and a trend change) was used to check for serially correlated errors by plotting the graphs of the residuals from the OLS regression (Fig. 2) as well as generating the autocorrelation and partial autocorrelation (ACF/PACF) plots. Significant and easily identifiable peaks at certain lags on the ACF/PACF plots was observed in 5 of the six variables as shown in Fig. 3.

**Fig. 4:**
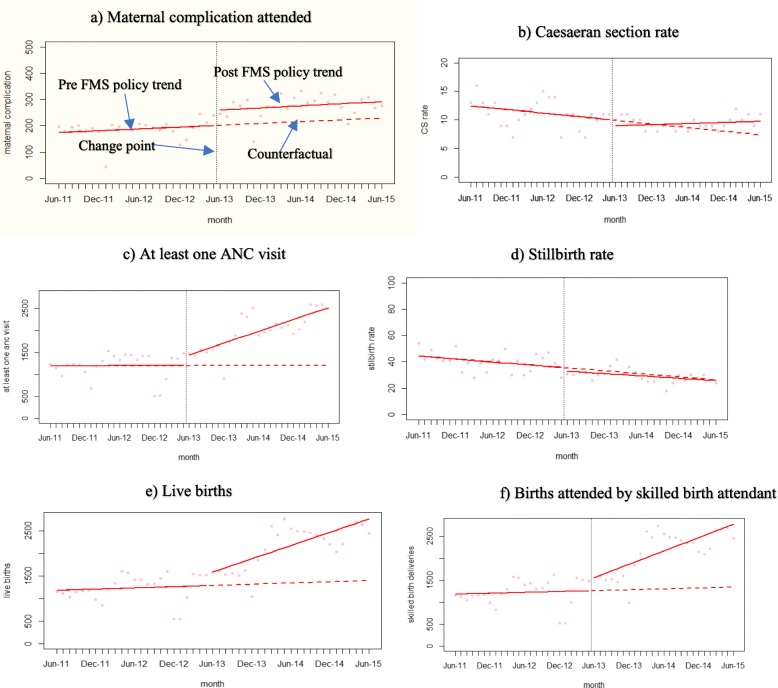
plots of time series of observed changes in the variables of interest. **a** Maternal complication attended. **b** Caesaeran section rate. **c** At least one ANC visit. **d** Stillbirth rate. **e** Live births. **f** Births attended by skilled birth attendant

Generalized least squares (gls) model, which fits a linear model with the respective autoregressive moving average process to adjust for autocorrelation, was used to run the results for each of the six variables. The results were then plotted with the counterfactual, which displays what the outcome would have been absent of the implementation of the free maternity policy (Figs. 4). Absolute and relative changes were calculated post policy implementation to meaningfully describe the effect of the policy.

The time series model took the following form;

For intervention status j, at time t:

$$ \mathrm{Outcome}\ \mathrm{j}\mathrm{t}=\upbeta 0+\upbeta 1\cdotp \mathrm{time}\ \mathrm{t}+\upbeta 2\cdotp \mathrm{level}\ \mathrm{j}+\upbeta 3\cdotp \mathrm{trend}\ \mathrm{j}\mathrm{t}+\upvarepsilon\ \mathrm{j}\mathrm{t} $$

β0- Represents the starting level of the outcome variable.

β1- Represents the slope or the trajectory of the outcome variable until the introduction of the intervention.

β2- Represents the change in the level of the outcome that occurs in the period immediately.

following the introduction of the Free Maternity Service Policy.

β3- Represents the difference between the preintervention and the postintervention slopes of the outcome variable.

Statistical significance for parameter estimation was set at α = 5% (*p ≤ 0.05).*

All our analyses were performed in R studio version 3.4.3 [31] with our data consisting of monthly aggregate data for all variables of interest.

## Results

A total of 82,962 women from Kilifi, Turkana and Wajir Counties were included in the study. The major assumption of the study was that the existing level and trend of the variables of interest would remain the same following the implementation of the Free Maternity Service Policy.

Table 3 depicts the difference in the standardized effect sizes (slope change and level change) prior and post the implementation of the Free Maternity Service Policy. The level change was positive and statistically significant for pregnant women identified with obstetric complication and attended at either level 5,4 or 3 health facilities. The mean score before and immediately after the implementation of the policy showed an estimated increase of 58 pregnant women identified with obstetric complication and attended as compared to before the policy was implemented. The absolute change after policy implementation being an average monthly number of 63 pregnant women identified with obstetric complication and attended more per month than would have been expected had the policy not been implemented. This represents a 27% increase.

**Table 3:** Parameter estimates from the gls regression model together with the 95% confidence interval

Parameters	Skilled birth delivery	Live births	Stillbirth rate	Women with at least one Antenatal visit	Caesarean rate	pregnant women identified with obstetric complication and attended in level 5,4,3 health facilities
Intercept pre-change point (β0)	1179*** (952.98,1406.14)	1177*** (954.711399.51)	44.87***(39.39,50.34)	1198.21*** (945.68,1450.73)	12.6*** (9.8, 15.38)	173.0***(11.93, 17.70)
Post intervention Level change (β2)	253.05 (− 101.24,607.35)	257.07 (−90.23, 604.37)	−2.55 (− 9.87,4.77)	201.04 (− 134.21,536.30)	−1.12 (− 3.94,1.68)	58.50(11.99, 105.03) **
Slope pre-change point (β1)	3.31(−13.70,20.32)	4.48 (−12.20, 21.17)	− 0.38 (− 0.76,0.00)	0.12(− 1748,17.71)	−0.1(− 0.29,0.08)	1.14 (1.26, 3.54)
Slope post-change point (β3)	47.31***(27.88,66.73)	44.46*** (25.36, 63.56)	0.08 (−0.45,0.61)	44.64***(20.41,68.81)	0.14(−0.15,0.42)	0.20(− 3.10, 3.49)

The asterisk, *, denotes statistical significance of *p* ≤ 0.05

The estimated trend/slope change post Free Maternity Service Policy implementation was positive and statistically different from zero for, births attended by skilled birth attendants, women with at least one antenatal care visit, and live births. This means that, for everyone month increase in time, there was an estimated increase in, 47 births attended by a skilled birth attendant, 44 women attending at least one ANC visit, and 44 live births after the implementation of the policy as compared to before the policy was implemented. The absolute change after Free Maternity Service Policy implementation indicated an average monthly increase of 1293 births attended by skilled birth attendants, 1183 women with at least one antenatal care visit, and 1235 live births more per month than would have been expected had the existing trend persisted. This represented a 97, 98, and 89% increase respectively.

The change in the stillbirth rate and the caesarean birth rate were found not to be statistically significant, meaning that absent of the policy, the existing trend in stillbirth rate and caesarean birth rate would not have changed.

The pre-and post-change point regression for all the six outcome variables together with the counterfactuals are plotted in Fig. 4 which demonstrates concurrence with those of the Table 3 above.

## Discussion

This study highlights the extent to which Free Maternity Service Policy in Kenya is associated with access, utilisation and quality of maternal health care services. Whereas a similar study has been conducted in Kenya, only three indicators were observed, skilled birth facility deliveries, maternal and neonatal mortality. A non-significant decrease in the maternal and neonatal mortality and a significant increase the skilled birth delivery was demonstrated [32]. This paper has gone further to assess six variables of the nine process indicators proposed by United Nations Children’s Emergency Fund (UNICEF) and World Health Organization (WHO) to be used in place of the impact indicators to monitor the effect of health care programs on maternal mortality [22, 23].

Increase in the antenatal care (ANC) coverage is positively associated with increase in use of skilled care during birth where [33] use of skilled care during birth has been linked with reduction in the risk of maternal and perinatal morbidity and mortality [4, 34] and improved perinatal survival [33].

The results presented here show that the Free Maternity Service Policy is associated with increase in number of live births, health facility deliveries, and women who were attended by a skilled care provider at least once during pregnancy.

The findings also indicate an association between Free Maternity Service Policy and access to emergency obstetric maternity care (EmONC), where a 27%increase in the number of pregnant women identified with obstetric complication in county referral hospitals, sub county hospitals and health centers were managed. The met need for EmONC was however not reported in this study, as the exclusion of private facilities and abortion cases attended would have led to skewed measurement of met need.

With the causative factor for maternal and perinatal mortality being closely linked, this study sought to establish to some extent the effect of the policy on quality of maternity care based on the readily available data in the facilities. Stillbirth rate, a proxy indicator for quality of care during labor and delivery, was used to assess if the policy may have influenced quality of maternal health care [35]. The results indicated that the stillbirth rate was already on a decline prior to policy introduction and the difference before and after the policy implementation was not statistically significant. Previous qualitative work on the Free Maternity Service Policy however indicated perceived negative impact of the policy on the quality of maternity care owing to the increased workload which was not matched with increase in human resource [13, 14]. However, while this study demonstrates no significant change in the rate of stillbirths, suggesting no association on the changes in quality of maternal health care to implementation of the policy, we acknowledge the fact that this indicator forms only one of the proposed 15 WHO indicators for quality of maternal health. Thus more work needs to be done to assess the quality of care using the proposed WHO indicators for quality of maternal health care which have been assessed for their feasibility in low- and middle-income setting. [36]. Unlike the study in Senegal where the caesarean birth rate significantly increased from 4.2 to 5.6% over a 1 year after the caesarean section fee was abolished, [37] in this study, a non-significant increase in the caesarean birth rate was observed post Free Maternity Service Policy implementation. The non-significant increase could be explained by the few (14) facilities that offer cesarean section within the whole of the three counties, all of which were included in the study, as well as the limited cadres permitted to conduct caesarean sections in Kenya. The Kenya health system delivery structures allows for caesarean section to only be conducted in the level 4, 5, and 6 health care facilities [20]. Seemingly this procedure by and large is conducted by obstetrician/ gynecologist, the medical officers [37] and more recently in 2008, the Ministry of Health allowed for the clinical officer’s reproductive health (CORH) to provide Reproductive Health Services including conducting caesarean sections. However, a study done in Kenya assessing the proportion of CORH carrying out emergency and invasive procedures demonstrated scarcity of information regarding their formal deployment, regulations and strategy and equally their contribution of CORH in emergency obstetric, and gynecological care was less pronounced, relatively smaller, and difficult to assess [32].

The use of ITSA in this study allowed for measuring of the effects of the policy in a real world setting as took into consideration the secular changes as part of the analytic process. Since the choice of study design was non-experimental, claiming causality is limited as there could be other possible explanation for the observed changes [25, 27, 28]. However, the use of a long time series, and multiple indicators enabled this study to provide compelling evidence on the effects of the policy on maternal health care service utilisation, accessibility and to some extent the quality of care.

The visual display of the plots and graphs from this analysis provides powerful and easy to understand results suitable for policy makers. Due to financial limitations, only few facilities were included in the sample posing a challenge to the generalizability of the study findings. Data quality was high in only 90 of the 127 health facilities leaving out the other 37 health facilities from the analysis. This introduced a limitation to the study as the findings reported are in the context of the included health facilities.

This study recommends further analysis be done with the data disaggregated according to the various demographic dimensions. This may help the policy makers understand better the factors that may influence the performance of the policy.

The abstract of this study has been published on the lancet [38].

## Conclusions

After 2 years of implementing Free Maternity Service Policy in Kenya, immediate and sustained increase in the use of skilled care during pregnancy and childbirth was observed. These findings suggest that the hospital cost is the main expense incurred by most women and their families whilst seeking maternity care services and a barrier to maternity care utilisation. Overall, Free Maternity Service Policy, as a health financing strategy, has exhibited the potential of realizing the full beneficial effects of maternal morbidity and mortality reduction in Kenya.

## Data Availability

All data that support the findings of this study are in the custody of Evaline Lang’at and are available on request.

## Abbreviations

DHIS2: District Health Information Systems
ITSA: Interrupted Time Series Analysis
KEPH: Kenya Essential Package of Health
KHSSP: Kenya Health Sector Strategic and Investment plan
MMR: Maternal Mortality Ratio
MOH: Ministry of Health
UNICEF: United Nations Children’s Emergency Fund
WHO: World Health Organization
